# Serratus anterior plane block in modified radical mastectomy surgery: a case series

**DOI:** 10.1186/s40981-020-00373-0

**Published:** 2020-10-15

**Authors:** Madonna Damayanthie Datu, Jokevin Prasetyadhi

**Affiliations:** 1 Department of Anesthesiology, Intensive Care, and Pain Management, Faculty of Medicine, Hasanuddin University/ RSUP Dr. Wahidin Sudirohusodo, Jl. Perintis Kemerdekaan KM. 11, Makassar, Tamalanrea 90245 Indonesia; 2 Faculty of Medicine, Hasanuddin University, Makassar, Indonesia

**Keywords:** Analgesia, Bupivacaine, Mastectomy, Nerve block, Pain, Serratus anterior plane block, Ultrasonography

## Abstract

**Background:**

Postoperative breast pain may lead to poorer outcome if left untreated. Common analgesia modalities for postoperative breast pain include opioids and regional anesthesia. However, both of these modalities can cause significant side effects or complications. Serratus anterior plane (SAP) block is a new procedure that is relatively easier to perform and safer, compared with other modalities. Previous studies have reported its usefulness in reducing the need for both intraoperative and postoperative opioids.

**Case:**

We reported 2 patients that underwent SAP block combined with general anesthesia in modified radical mastectomy (MRM). Patient 2 was given rescue analgesia during the intraoperative period. The administration of postoperative opioids did not exceed 24 h in both patients. Pain assessment using numeric rating scale (NRS) showed minimal postoperative pain. No side effects were found during 24-h monitoring period.

**Conclusion:**

SAP block can be used as one of the modalities in managing the pain of MRM surgery.

## Background

Mastectomy is a commonly performed surgery for breast malignancy. Around 10–20% of patients experience postoperative pain known as postmastectomy pain syndrome [1]. Untreated acute pain may develop into chronic pain (paresthesia, phantom breast pain, and intercostobrachial neuralgia) in 25–60% of cases, which led to poorer clinical outcome [2, 3]. Effective pain management is one of the crucial components in enhanced recovery after surgery (ERAS). Thus, the selection of appropriate analgesia modalities is needed [2].

Postoperative analgesia in mastectomy can be achieved by administration of opioids and regional anesthesia such as thoracic epidural block (TEB), paravertebral block (PVB), or intercostal blocks. Serratus anterior plane (SAP) block is an interventional technique that recently gained popularity in the context of breast surgery. SAP block resulted in better hemodynamic stability, early ambulation, and reduced duration of hospitalization as well as hospital costs in postoperative breast patients [4]. This case report highlighted the effectivity of SAP block in patients undergoing modified radical mastectomy (MRM) surgery.

## Case report

We reported 2 cases of women that underwent SAP block in MRM surgery.

### Case 1

**Fig. 1:**
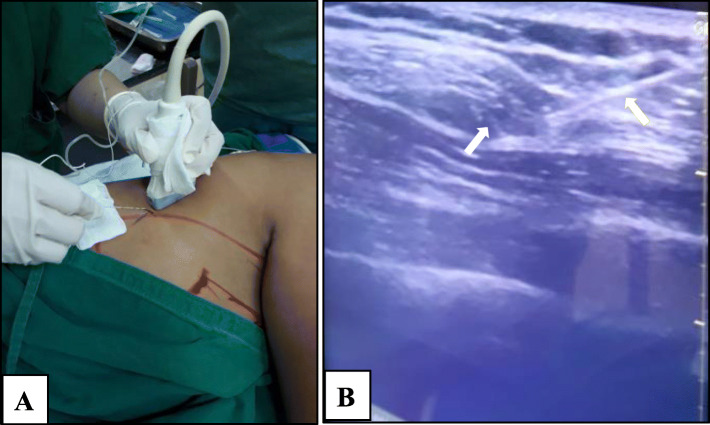
Serratus anterior plane block in patient 1. **a** Needle position, patient in lateral decubitus position. **b** Ultrasound image when injecting local anesthetic (yellow arrow = needle; white arrow = local anesthetic deposit)

**Fig. 2:**
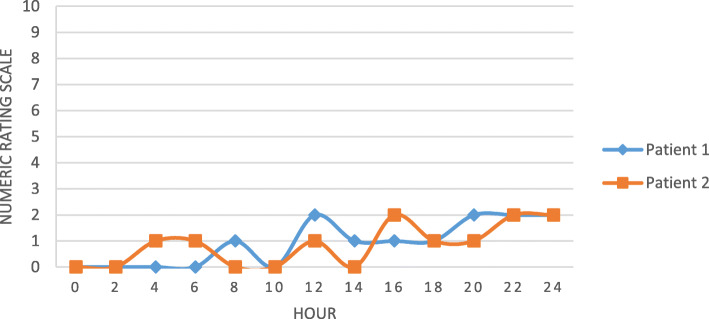
Postoperative numeric rating scale (NRS)

A 44-year-old woman (height 155 cm, body weight 65 kg) was diagnosed with right breast carcinoma and was scheduled to undergo right MRM surgery under general anesthesia and right SAP block. The patient had ASA physical status II, and preoperative examinations were unremarkable. The procedure was performed after informed consent for the SAP block was obtained. The procedure was performed under ultrasonography (USG) guidance using a 38-mm 6 MHz linear transducer and a 22G 100-mm regional block needle. The patient was in the left lateral decubitus position. Aseptic and antiseptic techniques were done around the area of mid-axillary line. Scans were performed while moving the transducer laterally and distally until the 4th and 5th ribs were visualized. The transducer was then rotated into the coronal plane and tilted posteriorly until the serratus anterior muscle and latissimus dorsi muscle were identified. After local infiltration with 1% lidocaine 2 ml and confirming no intravascular injection by aspiration, 0.25% bupivacaine 30 ml was injected on the fascia between the serratus anterior muscle and latissimus dorsi muscle (Fig. 1). For general anesthesia, patients were induced using 1% propofol 2 mg/kg with titration, isoflurane 1.5–2.0 vol%, and atracurium 0.1 mg/kg. The patient was intubated using direct laryngoscopy with endotracheal tube (ETT) no. 7. Ringer lactate was used as the maintenance liquid. The duration of the surgery was 120 min. Intravenous ketorolac 30 mg/8 h and oral paracetamol 500 mg/8 h were given as postoperative analgesia for 48 h. Intravenous tramadol 100 mg/8 h was also given for 24 h. The patient was monitored for 2 h postoperatively in recovery room, then for 24 h postoperatively in medical ward. Postoperative NRS is demonstrated on Fig. 2. The patient does not report any nausea/vomiting or breakthrough pain during monitoring period.

### Case 2

**Fig. 3:**
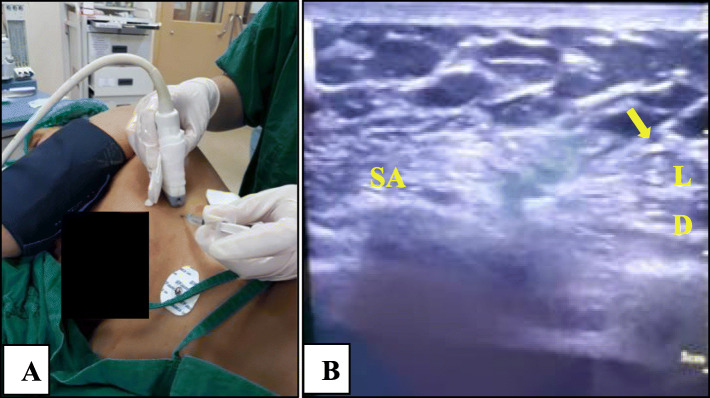
Serratus anterior plane block in patient 2. **a** Local infiltration. **b** Ultrasound image when injecting local anesthetic (LD, latissimus dorsi muscle; SA, serratus anterior muscle; yellow arrow, needle)

A 50-year-old woman (height 154 cm, body weight 63 kg) was diagnosed with left breast carcinoma and was scheduled to undergo left MRM surgery under general anesthesia and left SAP block. The patient had ASA I physical status classification. Preoperative examinations were unremarkable. Left SAP block was performed in left lateral decubitus position. SAP block steps were similar as case 1 (Fig. 3). The patient was also given a similar protocol as case 1 for general anesthesia. Fifteen minutes after initial incision, an increase in heart rate and mean arterial pressure (MAP) of > 20% was noticed. The patient was given 30 μg of intravenous fentanyl as rescue analgesic. The duration of surgery was 140 min. The patient received similar postoperative analgesics and monitoring as the patient in case 1. There was also no marked side effect or breakthrough pain during monitoring period.

## Discussion

SAP block is one of the newest thoracic regional anesthetic techniques. This procedure is an alternative to TEB, PVB, intercostal blocks, and intrapleural blocks that are commonly used as postoperative analgesia of the thoracic region [5]. Anatomically, SAP block was performed in the superficial plane which was considered safer than other regional anesthetic techniques [6]. SAP block is indicated in conditions that require pain management in the thoracic region, such as rib fractures, rib contusions, thoracoscopic surgery, thoracotomy, breast surgery, and post-mastectomy pain syndrome [7].

SAP block exerts its analgesic effects on the lateral thoracic region [8] This effect is achieved by nerve blockade in the axillary fossa which includes the intercostobrachial nerve, the cutaneous intercostal nerve (T3-T9), the thoracic longus nerve, and the thoracodorsal nerve located in the compartment between the serratus anterior muscle and latissimus dorsi muscle, and between the posterior and mid-axillary lines [9]. The main anatomical landmarks in the SAP block are the latissimus dorsi muscle and serratus anterior muscle. The thoracodorsal arteries run in the fascia plane between these two muscles [10]. Using USG, the anterior serratus muscle appears as a thick hypoechoic appearance below the latissimus dorsi muscle and above the rib cage [5]. To date, there are still no definitive criteria for optimal volume and concentration for local anesthetic in SAP blocks [2]. In this study, SAP block was performed using roughly 0.4 ml/kg of long-acting local anesthetic.

Blanco et al. studied the effectivity of SAP blocks with USG in 4 volunteers [3]. The authors found 2 types of space for injection, which were superficial and deep. The distribution of dermatomes in superficial SAP block tends to be wider than deep SAP than SAP, which is T3-T9 [3]. Bhoi et al. found that pain levels were lower in the superficial SAP block group than in the deep SAP block group. This finding may be due to the effect of long thoracic nerve and thoracodorsal nerve blockade in superficial SAP blocks [6]. Both patients in this case underwent superficial SAP block. Patient 2 underwent surgery with a particularly high incision, reaching T2 level. The incision might trigger the pain that required rescue analgesia since the intraoperative pain stimulus has exceeded the dermatomal level of the SAP block, which was T3.

SAP blocks are relatively easy procedure with a high success rate and minimal complications when performed by a trained anesthetist. SAP blocks usually only require one injection, whereas most of other regional blocks require repeated injections [11]. The analgesia effect produced by the SAP block may last up to 12 h postoperatively [4]. Semyonov et al. found that patients who underwent SAP block had significantly lower pain levels after thoracic surgery, compared to patients in the standard pain control group [11]. The total dose of morphine and tramadol needed to relieve pain during the first few postoperative hours is significantly lower in patients that underwent SAP block. The incidence of side effects such as nausea and vomiting is significantly lower as well [11]. Chen et al. demonstrated that SAP blocks provided superior analgesic effect, where pain scores and opioid consumption were significantly lower in the postoperative period [2]. Either of our patients did not require intraoperative opioid use, except when given as rescue analgesia in patient 2. Intravenous tramadol 100 mg/8 h was given as postoperative analgesics in both patients, and not exceeding 24 h. The duration of the analgesia effect of the SAP block is consistent with literature, where both patients have a minimum NRS and only begin to increase 12 h after the surgery or 14 h after the block was performed. The duration of this effect was also influenced by other analgesics. In both patients, there were no side effects up to 24 h postoperatively. This finding was expected since opioid administration was minimized. The results of these two cases suggested the need of clinical investigations focusing on opioid consumption and postoperative unpleasant symptoms and recovery.

In conclusion, SAP block can be used as one of the modalities in managing the pain of patients undergoing MRM surgery. This procedure was effective in reducing the need for both intraoperative and postoperative opioids usage.

## Data Availability

Not applicable

## Abbreviations

ECG: Electrocardiography
ERAS: Enhanced recovery after surgery
ETT: Endotracheal tube
MAP: Mean arterial pressure
MRM: Modified radical mastectomy
NRS: Numeric rating scale
PVB: Paravertebral block
SAP: Serratus anterior plane
TEB: Thoracic epidural block
USG: Ultrasonography
